# Mechanical and Biological Properties of Nondecellularized Human Wharton's Jelly Matrix for Soft Tissue Graft Material: An *In Vitro* Study

**DOI:** 10.1055/s-0045-1809981

**Published:** 2025-07-23

**Authors:** Mefina Kuntjoro, Nike Hendrijantini, Eric Priyo Prasetyo, Nurani Atikasari, Made Shintya Danaswari, Guang Hong

**Affiliations:** 1 Department of Prosthodontic, Faculty of Dental Medicine, Universitas Airlangga, Surabaya, Indonesia; 2 Department of Conservative Dentistry, Faculty of Dental Medicine, Universitas Airlangga, Surabaya, Indonesia; 3 Division for Globalization Initiative, Liaison Center for Innovative Dentistry, Graduate School of Dentistry, Tohoku University, Aoba-Ku, Sendai, Japan

**Keywords:** tissue engineering, regeneration, allograft, freeze drying, medicine

## Abstract

**Objective:**

This study aims to determine the mechanical properties of hWJM through ultimate tensile strength (UTS) and elastic modulus tests, degradation, and porosity tests. Additionally, it evaluates the biological properties of hWJM by assessing growth factor secretion (fibroblast growth factor-2 [FGF-2] and vascular endothelial growth factor-A [VEGF-A]).

**Materials and Methods:**

For the mechanical tests, two groups were analyzed: group 1 was tested in a wet condition and group 2 in a dry condition. Degradation tests were conducted using phosphate buffer saline (PBS), collagenase enzyme, and simulated body fluid (SBF). The porosity test was conducted using a scanning electron microscope (SEM). For the biological tests, two groups were analyzed: group 1 consisted of nondecellularized hWJM and group 2 of human umbilical cord mesenchymal stem cells. All the data were collected and analyzed statistically.

**Results:**

The mean ± standard deviation of the UTS test for group 1 was 0.787 ± 0.356 MPa, while for group 2, it was 1.897 ± 0.582 MPa. The elastic modulus test results were 0.568 ± 0.206 MPa for group 1 and 6.354 ± 2.985 MPa for group 2. The result showed that PBS degradation was 22% on day 14 and 39% on day 28. The collagenase enzyme degraded 15% after 1 hour, 63% after 6 hours, and 74% after 24 hours. SBF degradation rates were 10.3% after 1 week, 10.5% after 2 weeks, 12.5% after 4 weeks, and 13.9% after 8 weeks. The porosity test results showed an average pore size of 66.95 μm. For the biological tests, no significant differences in FGF-2 and VEGF secretion were observed between groups, with the highest secretion in group 1 occurring on day 7.

**Conclusion:**

Nondecellularized hWJM has been shown to meet the physical, mechanical, and biological criteria for soft tissue graft in dentistry.

## Introduction

Soft tissue graft for tissue engineering is a crucial requirement in the field of dentistry, particularly in prosthodontic or oral surgery cases such as implant placement that requires guided bone regeneration or guided tissue regeneration (GTR), as well as in periodontics cases such as flap surgery for gingival recession. Tissue engineering in regenerative dentistry focuses on restoring, repairing, or replacing damaged cells and tissues using stem cells, growth factor, and scaffolds. These scaffolds or graft may function as acellular structures or be integrated with living cells. 1 Ongoing advancements in tissue engineering continue to identify new candidates for tissue-engineered products.

Three primary types of soft tissues are: autogenous grafts, allografts, and xenografts. 2 Among these, autogenous connective tissue grafts (CTGs), particularly subepithelial CTGs (SCTGs) harvested from the palate, retromolar pad, or edentulous areas, are considered highly effective in improving gingival health. However, SCTGs have certain limitations, including donor site morbidity, limited tissue availability, and extended procedure times for graft harvesting and placement. 3 The use of acellular dermal matrix (ADM) soft tissue allografts derived from human donors carries a potential risk of disease transmission. 4 Moreover, studies have indicated that CTG offers superior outcomes compared with ADM in procedures involving GTR, as ADM has been associated with secondary gingival recession and a reduction in keratinized soft tissue (KST) thickness. 5 Xenografts used for soft tissue have the disadvantage of lacking cellular elements and do not contain the growth factors necessary to stimulate cytokines required for increasing KST thickness. 6 Continued innovation in biomaterials is being pursued to effectively address these concerns.

Effective soft tissue graft biomaterials are expected to meet two fundamental standards, which are mechanical and biological properties that support the processes of modeling and remodeling and volume stability over a certain period. 7 Graft materials should have mechanical properties similar to the original tissue to restore physiological function in the human body, which meets several criteria, including high porosity, biocompatibility, and biodegradability. 8 For biological standards as a scaffold, grafts must integrate with host tissue and provide a suitable environment for cell growth and differentiation. 9 The vascular endothelial growth factor-A (VEGF-A) and fibroblast growth factor-2 (FGF-2) were investigated as their physiologic functions in tissue healing and angiogenesis, as well as a multipurpose biomolecules controlling cell proliferation and adhesion. 10

Wharton's jelly (WJ) is a surgical byproduct but has strong biocompatibility, low immunogenicity, and positive ethical implications due to its extracellular matrix (ECM) components, such as collagen, glycosaminoglycans, and growth factors. Human Wharton's jelly matrix (hWJM) is one of the most promising allograft materials currently under development. 11 There are two main methods for preserving WJ: cryopreservation and freeze-drying. 11 12 Cryopreservation is the most commonly used preservation technique; however, it has certain drawbacks. It requires storage at extremely low temperatures, making both storage and application challenging. 12 In contrast, freeze-drying offers more flexibility in usage timelines and allows storage at room temperature, making handling more convenient. 11 Additionally, it possesses adequate physical and mechanical properties to serve as a scaffold for stem cell delivery. 13

Research on WJ has predominantly focused on decellularized scaffolds. The decellularization process effectively eliminates antigenicity and immunogenic responses to tissues and organs. 14 However, it also removes bioactive components, such as growth factors, which are critical for promoting regeneration in tissue engineering applications. 15 To evaluate the suitability of soft tissue graft candidates, various mechanical and biological properties must be assessed. These include tensile strength and degradation tests, as well as surface morphology analyses to determine stem cell adhesion and proliferation. Such evaluations are essential for understanding the biological properties of scaffolds and their potential to support tissue engineering products.

## Materials and Methods

### Freeze-Dried hWJM Preparation

WJ was extracted from the human placenta, which is collected from preselected donor after the cesarean delivery. Following the collection, the placenta is stored in a sterile container immersed in the transport medium, which consists of α-minimum essential medium, fetal bovine serum, and Fungizone, and then transported for processing. The transport time should be as short as possible, with a maximum time of 24 hours. Prior to preparation, the WJ is washed sequentially with glycerol, saline, and aquades. The sample was divided into two parts: one for human umbilical cord mesenchymal stem cells (hUCMSCs) and the other for use as hWJM. After stretching, the WJ designated for scaffold samples underwent lyophilization or freeze-drying to remove its water content. This process involved freezing the sample, followed by sublimation drying, resulting in freeze-dried WJ. It was then cut into 2 cm × 2 cm pieces and sterilized using gamma irradiation at a dose of 25 kGy. After that, the hWJM was stored at room temperature.

### hUCMSCs Preparation

The procedure was done according to a previous study. 16 For mesenchymal stem cell (MSC) confirmation, flow cytometry was performed using primary antibodies against human CD73, CD90, and CD105, along with a negative cocktail (CD45, CD34).

### Ultimate Tensile Strength and Elastic Modulus Test

The hWJM was divided into two groups: group 1 manipulated in a wet condition and group 2 in a dry condition. 17 The thickness of WJM was measured using a micrometer screw gauge or a thickness gauge with an accuracy of 0.01 mm. Measurements were taken at three random points on each sample. The hWJM samples were shaped into rectangular specimens with dimensions of 20 × 10 × 1.5 mm. Some samples were rinsed and soaked in a phosphate buffer saline (PBS) solution for 2 hours. The membranes were then secured at both ends in a jig within a universal testing machine and tested at a crosshead speed of 0.05 mm/s.

### Degradation Test Using PBS, Collagenase Enzyme, and Simulated Body Fluid

The prepared samples were square-shaped with dimensions of 10 × 10 × 1.5 mm. Each group consist of three samples according to previous research. 18 The initial weight of the samples (W _0_ ) was measured. For PBS test, the samples were immersed in Roux bottles containing PBS solution at pH 7.4 and maintained at a temperature of 37°C for 14 days. Afterward, the membranes were removed, rinsed with sterile distilled water, frozen at –80°C, and lyophilized for 2 × 24 hours. The dried samples were then weighed to determine their final weight (W _t_ ). This procedure was repeated with an immersion duration of 28 days. 19 For collagenase enzyme test, samples was immersed in a 6-well plate at a concentration of 2 IU/mL in 0.05 M Tris-HCl (pH 7.4) with 0.01 M CaCl _2_ at 37°C for 1 hour. The sample was then removed, rinsed with sterile distilled water, frozen at –80°C, and lyophilized for 48 hours. The dried sample was then weighed to determine the final weight (W _t_ ). This procedure was repeated with immersion durations of 6 and 24 hours. For simulated body fluid (SBF) test, samples were placed in a 6-well plate, and 5 mL of SBF was added to each well. The plate was incubated in an orbital shaker at 37°C for 1, 2, 4, and 8 weeks. Every week, 5 mL of SBF was replaced with a fresh solution. After the designated incubation periods, the samples were removed from the fluid, transferred to a −80°C freezer for 24 hours, and then freeze-dried to eliminate any remaining moisture. The dry weight of each sample (W _1_ ) was recorded. 20

### Porosity Test Using Scanning Electron Microscope

The scanning electron microscope (SEM) holder was prepared by attaching double-sided carbon tape. One side of the tape was adhered to the SEM holder, while the other side was used to affix the hWJM scaffold for examination. The coating was performed using a sputter coater (Quorum SC7620) by placing the membrane and holder into the sputter coater, followed by vacuuming for 10 minutes. After vacuuming, plasma coating was performed for 10 seconds using platinum. The coated sample was then placed into the holder on the SEM (Hitachi TM3000). The sample underwent a vacuum process for 5 minutes, after which images appeared on the monitor. Images were captured at magnifications of 1000× at a voltage of 20 kV. The SEM micrographs were converted to binary images and analyzed using the ImageJ software to evaluate the pore size of the matrix.

### FGF-2 and VEGF-A Growth Factor Secretion Using Enzyme-Linked Immunosorbent Assay

The secretion of growth factors was assessed in hWJM scaffolds for group 1 and in hUCMSCs for group 2. Both groups were evaluated on days 1, 3, 7, and 10. The culture medium was replaced every 3 days, collected and accumulated until the observation days. The secretion of VEGF-A and FGF-2 was examined using the enzyme-linked immunosorbent assay (ELISA) method with human VEGF-A and human FGF-2 kits (Bioassay Technology [BT] Laboratory, China). The procedure began with running the standard curve. All samples and reagents were added to the respective wells and incubated for 1 hour at 37°C. Solution A and solution B were then added, followed by an additional 10-minute incubation. A stop solution was added, and color development was observed, with the optical density read within 10 minutes. Measurements were performed according to the ELISA protocol provided by the manufacturer. The measurement ranges for ELISA were as follows: VEGF-A: 3 to 900 ng/L and FGF-2: 5 to 1500 ng/L.

## Results

### Ultimate Tensile Strength and Elastic Modulus Test

**Fig. 1:**
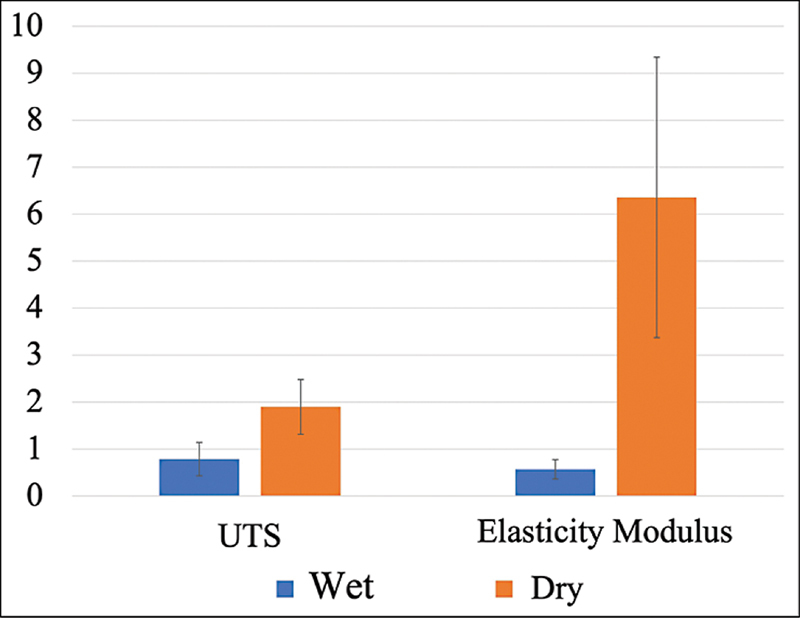
Ultimate tensile strength evaluation and elastic modulus test on group 1 (wet condition indicated by blue color) and group 2 (dry condition indicated by orange color).

For sample group 1, the mean and standard deviation of the ultimate tensile strength (UTS) were 0.787 ± 0.356 MPa, with an elastic modulus of 0.568 ± 0.206 MPa. In contrast, sample group 2 showed a UTS value of 1.897 ± 0.582 MPa and an elastic modulus of 6.354 ± 2.985 MPa. These data sets were analyzed using SPSS, revealing that both data groups were normally distributed and homogeneous. The UTS and elastic modulus values indicated a statistically significant difference ( *p* < 0.05), with sample group 2 exhibiting higher values than group 1 ( Fig. 1 ).

### Degradation Test Using PBS, Collagenase Enzyme, and SBF

**Fig. 2:**
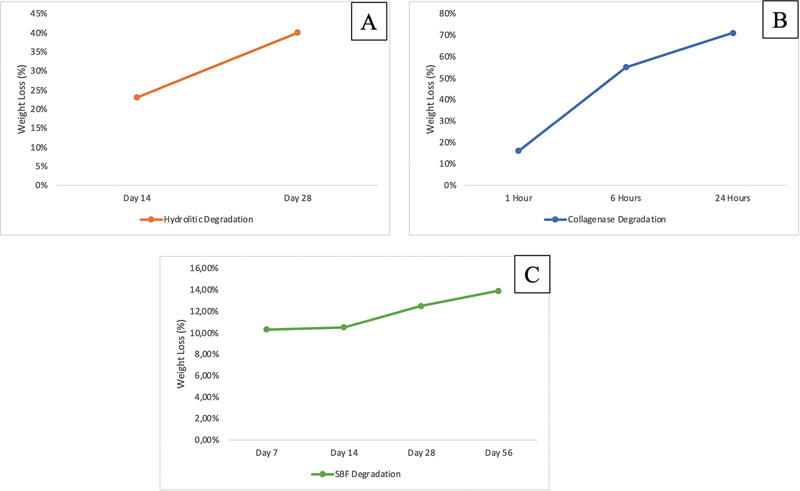
Degradation test evaluation of nondecellularized human Wharton's jelly matrix (hWJM) ( **A** : using phosphate buffer saline [PBS]; **B** : using collagenase enzyme; **C** : using simulated body fluid [SBF]).

Fig. 2A presents hydrolytic degradation rate tests, with observations conducted at two time points: day 14 and day 28. The average hydrolytic degradation rate was 22% on day 14 and 39% on day 28.

Fig. 2B presents data from collagenase enzyme degradation rate tests observed at three time intervals: 1, 6, and 24 hours. The means of degradation rate in SBF were 15, 63, and 74%, respectively.

Fig. 2C shows the degradation in SBF, which followed a progressive trend over time. After 1 week, degradation was recorded at 10.3%, increasing slightly to 10.5% after 2 weeks. As exposure time extended, the degradation rate rose to 12.5% after 4 weeks and 13.9% after 8 weeks.

### Porosity Test Using SEM

**Fig. 3:**
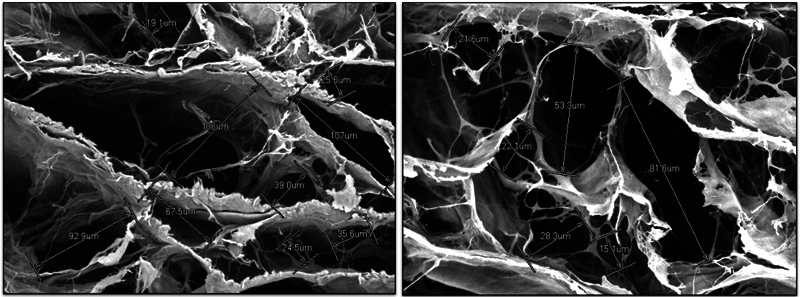
Porosity test analysis of human Wharton's jelly matrix (hWJM) using scanning electron microscope (SEM) captured by the ImageJ software with magnification 1000× showed the pore size of hWJM.

Surface porosity analysis of the hWJM scaffold was conducted by measuring 20 randomly selected pores on each sample using the ImageJ software. Quantitative data revealed that the average pore size of the hWJM samples in this study was 66.95 μm, with pore sizes ranging from 34.80 to 103.90 μm ( Fig. 3A , B ).

### FGF-2 and VEGF-A Growth Factor Secretion Using ELISA

**Fig. 4:**
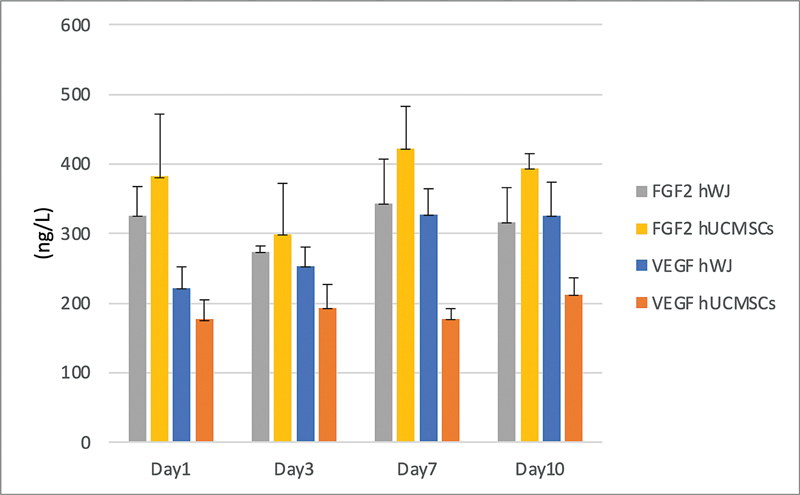
The growth factor secretion (fibroblast growth factor-2 [FGF-2] and vascular endothelial growth factor-A [VEGF-A]) between human Wharton jelly matrix (hWJM) groups and human umbilical cord mesenchymal stem cells (hUCMSCs) groups using enzyme-linked immunosorbent assay (ELISA).

FGF-2 and VEGF-A secretion by hWJM for group 1 and hUCMSCs for group 2 in the medium was initiated during passage 5. Each medium color change was observed and examined using an ELISA reader. The media were collected once daily on days 1, 3, 7, and 10. In group 1, FGF-2 secretion peaked on day 7, followed by a decline on day 10. Similarly, in group 2, FGF-2 secretion peaked on day 7 before decreasing on day 10. In contrast, VEGF-A secretion in group 1 peaked on day 7, while in group 2, it peaked on day 10. The corresponding data can be seen in Fig. 4 .

## Discussion

Tissue engineering aims to develop tissue analogs that support cellular activities crucial for the regeneration of lost or damaged tissues and organs. Scaffolds provide a structural framework for cell attachment and must permit adequate cell infiltration to promote functional tissue formation. Consequently, it is essential to design biocompatible scaffolds—whether from native human tissue, synthetic materials, or their composites—that closely replicate the properties of the ECM. 8 10 11 The hWJM, a mucous connective tissue derived from the umbilical cord, is characterized by its high molecular weight and abundance of ECM such as collagen, hyaluronic acid, and proteoglycans. 21

One of the most important characteristics for a biomaterial as a scaffold is its ability to exhibit mechanical properties similar to those of native tissue. 22 A common method for evaluating the mechanical properties are UTS and elastic modulus. The UTS determines a material's mechanical resistance to deformation and rupture under various pressures, while the elastic modulus controls its ability to return to its original shape after an external force is removed. Both parameters play a crucial role in determining a material's degradation behavior, cellular interactions, tissue repair, and regenerative capacity in medical applications. 23

In this study, the mechanical properties of hWJM were evaluated in both dry and wet states. Dry hWJM exhibited higher mechanical strength than wet hWJM, with UTS values of 1.9 ± 0.6 MPa versus 0.8 ± 0.4 MPa and elastic moduli of 6.4 ± 3.0 MPa versus 0.6 ± 0.2 MPa, respectively. These findings align with those of Raz et al, who observed higher mechanical properties in dry collagen membranes. Similarly, Bose et al reported a reduction in UTS and the development of hyperelastic characteristics when collagen membranes were immersed in liquid, attributing this to the absorption of water molecules, which caused collagen fibrils to slide and increased elasticity. 24

Previous studies have reported that the UTS of gingival mucosa is approximately 3.8 MPa, with an elastic modulus of 37.4 MPa. Furthermore, Choi et al examined the mechanical characteristics of buccal mucosa, reporting a UTS of 1.5 MPa and an elastic modulus of 8.3 MPa. 25 Based on the results, the mechanical properties of dry hWJM are comparable to those of buccal mucosa but lower than those gingival mucosa. This indicates that hWJM possesses sufficient mechanical strength for soft tissue management in implant procedures. However, upon hydration, hWJM exhibits reduced mechanical strength, which may compromise its structural integrity under physiological conditions, potentially affecting tissue healing and periodontal regeneration. Future research should focus on enhancing the mechanical strength of hWJM or developing protective devices to shield the material from mechanical forces in the oral environment.

An ideal scaffold should degrade at a rate that aligns with tissue regeneration; if degradation occurs too rapidly, it can lead to early mechanical weakness, compromising structural support. Conversely, if degradation is too slow, it may provoke an immune response, leading to fibrous encapsulation. One key factor determining degradation rate is the biomaterial's surrounding environment, including factors such as humidity and enzyme presence. 26 The presence of water and collagenase enzymes produced by periodontopathogenic bacteria beneath the soft tissue structure substantially impacts the scaffold's breakdown and integration with the surrounding tissue. 27

A previous study investigated the degradation rates of three collagen matrices used in soft tissue augmentation: Fibro-gide, Mucograft, and Mucoderm. The hydrolytic degradation rates at h + 14 and h + 28 days were 68 and 77%, 93 and 100%, and 100 and 100%, respectively. The hWJM shows superior hydrolytic degradation (22 and 39%) compared with the three collagen matrices. The degradation rate of GTR membranes is crucial for ensuring that collagen matrices are successfully incorporated and accepted by the recipient tissue. It also plays a key role in maintaining adequate volumetric stability, thereby providing sufficient time for cell and blood vessel infiltration. It was concluded that 14 days would provide adequate time for cell infiltration and revascularization, and that the matrix should remain intact for a minimum of 4 weeks to support the incorporation and regeneration of oral tissues. 28

For evaluating the breakdown of collagen membranes, collagenase solutions derived from *Clostridium histolyticum* are considered highly aggressive media. They are regarded as a suitable test for membranes exposed to worst-case conditions, such as wound dehiscence or open wounds. 1 9 We compared the results obtained from hWJM with three matrices commonly used as materials for soft tissue regeneration (Fibro-gide, Mucograft, Mucoderm). The collagenase enzymatic degradation rates of the three materials at h + 1, h + 6, and h + 24 hours were as follows: −22, 17, and 64% for Fibro-gide®, 12, 89, and 98% for Mucograft, and −2, 16, and 60% for Mucoderm. Based on the above results, Mucoderm demonstrates resistance to the exposed oral environment and is recommended for open healing cases. Compared with this study, hWJM degradation rates which are 15, 63, and 74% demonstrates comparable resistance to Mucoderm. Therefore, it holds potential for clinical applications such as recession coverage and open healing procedures. 28

The ionic species in the concentrated solution altered the solubility of collagen and affected its mechanical properties, simulating the blood plasma ion concentration. The use of SBF revealed that collagen scaffolds break down and become less stable within 14 days, whereas human blood plasma showed no substantial mechanical changes or degradation during that time. Besides, this phenomenon may be attributed to the measurement methodology employed, which involved assessing the dry weight of the samples. Some research shows that certain biomaterials can exhibit mass increases due to the absorption of ions or the formation of mineralized layers on their surfaces when immersed in SBF. 29 For instance, it is reported that materials like polycaprolactone composites and silk fibroin fibers can undergo surface mineralization, leading to an apparent increase in mass during SBF immersion. 30 Clearer results were obtained with PBS, where mechanical stability decreased throughout the first 7 days before stabilizing, suggesting a slow decline that should not persist. 31 This stable biodegradability offers significant advantages in periodontal regeneration by eliminating the need for a second surgical procedure.

Based on the data presented above regarding UTS and elasticity, it can be concluded that hWJM exhibits certain limitations in these mechanical properties compared with native. However, its degradation rate is comparable to that of other biomaterials currently available in the markets. As the mechanical strength increases, the slower degradation rate becomes a limitation; conversely, when a biomaterial exhibits favorable degradation, its mechanical strength tends to be lower. 31 Slower degradation rate can be advantageous for long-term structural support but may hinder tissue integration over time. On the other hand, materials with favorable degradation rates may offer better integration with surrounding tissues but at the cost of reduced mechanical strength. In summary, the degradability of biodegradable materials significantly influences both parameters, thereby affecting cellular interactions, tissue repair, and regenerative capacity in medical applications. 25 According to this, the selection of the graft site and the graft material is crucial, as each region of the oral cavity requires specific biomechanical properties tailored to its distinct functional role. 32

In consideration to enhance mechanical strength and degradation rate of collagen matrices, cross-linking can be a valuable consideration for further research. Studies have demonstrated that cross-linked collagen matrices exhibit increased resistance to enzymatic degradation and mechanical stress compared with their noncross-linked counterparts. Therefore, incorporating cross-linking into collagen matrices may offer improved mechanical properties and prolonged degradation profiles, making them suitable for applications requiring sustained structural support. It is important to weigh these advantages against possible drawbacks, such as elevated inflammatory responses or foreign body reactions. To improve cross-linking techniques and evaluate their long-term impacts in diverse clinical settings, more study is required. 33

The macrostructure of a soft tissue graft plays a crucial role in determining its functionality and effectiveness in tissue engineering. The hWJM samples in this study was 66.95 μm, with pore sizes ranging from 34.80 to 103.90 μm, which fall into medium structures (20–40 μm) and large scale structures (> 100 μm). Medium structure regulates the polarization of macrophage 1 (M1) to macrophage 2 (M2) and enhances the expression of anti-inflammatory genes to modulate the host immune response to grafts, thereby promoting the infiltration of host cells, particularly MSCs. Large-scale structures promote angiogenesis, induce cell homing, offer a colony site, and formation. 34 With this range of pore sizes, hWJM also serves as a biomaterial that can provide a suitable environment for MSCs ranging in size from 20 to 40 μm to adhere and ensuring stable cell fixation on the scaffold. 13

This study demonstrates that hWJM is capable of secreting critical growth factors, including VEGF and FGF. Angiogenesis is critical in regenerative processes, as it ensures the necessary blood supply to newly formed or repaired tissues, providing oxygen and nutrients while removing waste products. In the context of soft tissue grafts, effective angiogenesis is paramount for the survival, integration, and functionality of the graft within the host tissue. Without adequate vascularization, grafted cells would suffer from ischemia and necrosis, leading to graft failure. VEGF-A is a key signaling protein and growth factor that promotes specific functions in vascular endothelial cells. It is widely used in tissue engineering for the functionalization of biomaterials. 13 FGF-2, also known as basic FGF, is a signaling molecule that plays a significant proangiogenic role by promoting the secretion of matrix metalloproteinases and regulating the proliferation, differentiation, migration, and maturation of endothelial cells, which is essential for new blood vessel formation and tissue regeneration. Moreover, VEGF-A and FGF-2 act as multifunctional biomolecules governing cell adhesion and proliferation, which is potential for tissue engineering materials. 10

Based on our findings, the release profiles of FGF-2 and VEGF-A from the biomaterial peaked on day 7, aligning with anticipated therapeutic timelines. However, prolonged release of growth factors may lead to adverse effects, such as receptor desensitization and altered cell signaling pathways. Given that the material's mechanical properties currently limit its application to soft tissue contexts, enhancing its strength through polymer incorporation and crosslinking techniques has been explored and widely applied. Furthermore, as the total amounts of FGF-2 and VEGF-A prior to material processing were not measured, the efficiency of growth factor retention remains undetermined. This aspect warrants further investigation in subsequent studies.

Within this result, nondecellularized ECM matrices offer distinct advantages in tissue engineering by retaining living cells that actively contribute to tissue regeneration through the secretion of bioactive factors and modulation of immune responses. Thus, it also maintains the native cellular composition and microenvironment of the tissue, including the ECM components and cell-to-cell interactions. This preservation ensures that the scaffold closely mimics the natural tissue structure, providing a more conducive environment for cell attachment, migration, and differentiation. The presence of living cells allows for dynamic responses to environmental cues, facilitating *in situ* tissue regeneration. These matrices can adapt to the healing site, promoting tissue repair and integration without the need for extensive recellularization processes. 35

The MSCs contained in the hWJM secreted various growth factors, cytokines, hyaluronic acid, and extracellular vesicles, all of which contribute to its potential as an effective biomaterial for regenerative dentistry. Stem cells from umbilical cord retained anti-inflammatory properties and antibacterial activity, which is crucial for graft material. *In vitro* and *in vivo* studies, both confirm positive result of MSCs as a cell-based therapy in dentistry. 36 37 38 39

## Conclusion

Nondecellularized hWJM has been shown to meet the physical, mechanical, and biological criteria for allograft soft tissue in dentistry. Despite its lower strength compared with autologous tissues, hWJM remains a promising alternative material for periodontal regeneration due to its favorable biological properties. However, this research is preliminary to find allograft biomaterial for use as soft tissue graft. More studies are necessary to validate these findings and optimize the material's performance in clinical settings. Given the preliminary nature of these findings, further *in vivo* studies are essential to validate the observed effects and establish the material's safety and efficacy in a living organism.
